# The Reformed Public House

**Published:** 1924-04

**Authors:** D. C. Dering

**Affiliations:** 76 Teignmouth Road, Cricklewood, N.W. 2


### CORRESPONDENCE.

[Correspondence on all subjects is invited, but we cannot be responsible for the opinions expressed by our correspondents, who must give name and address as a guarantee of good faith, but not necessarily for publication. Correspondents are reminded that conciseness greatly facilitates early insertion.]

### The Reformed Public House.

*To the Editor of* THE HOSPITAL AND HEALTH REVIEW.

SIR,—Speaking for myself, I am not acquainted with the views held by Mr. Grubb. I am not disposed to accept Mr. Simons's interpretation of my wishes in regard to licensing reform. The licensed trade is already a monopoly—a private monopoly granted by the State. My contention is that the holders of that monopoly have failed to keep their part of the bargain, having to a very large extent confined the business to a trade in beer to the neglect of their obvious duty to the community, which requires a real public house in its widest sense. This has been the inevitable result of a monopoly which enriches individuals at the expense of the people. As has already been explained in this correspondence, the Trade could provide the public house required, as the People's Refreshment House Association does, and still make a respectable profit on the buiness. There is no need for a Public House Improvement Bill to secure this. Such a Bill is designed to increase facilities for doing more trade in beer.

I, too, want to see the public house decent and comfortable, but I am sceptical as to this being effected by the brewer and the publican under the tied house system. Mr. Pace misrepresents the objects of the People's Refreshment House Association. True, it is a limited liability company, but it is governed by rules designed to secure disinterested management; the amount of interest to shareholders is fixed, and the managers of the houses are paid a fixed salary with no commission on the sales of alcoholic liquors, but only on the sales of food and non-intoxicants. Surplus profits are in some cases devoted to objects of public utility.

D. C. DERING.

76 Teignmouth Road,
Cricklewood, N.W. 2.

